# Checking Inhaler Technique in the Community Pharmacy: Predictors of Critical Errors

**DOI:** 10.3390/pharmacy8010006

**Published:** 2020-01-07

**Authors:** Tatiana Makhinova, Brandie L. Walker, Marlene Gukert, LeAnna Kalvi, Lisa M. Guirguis

**Affiliations:** 1 Faculty of Pharmacy and Pharmaceutical Sciences, University of Alberta, Edmonton, AB T6G 1C9, Canada; tatiana.makhinova@ualberta.ca (T.M.); mgukert@ualberta.ca (M.G.); kalvi@ualberta.ca (L.K.); 2 Department of Medicine, Division of Respirology, University of Calgary, Calgary, AB T2N 1N4, Canada; blthorla@ucalgary.ca

**Keywords:** inhaler technique, inhaler error, community pharmacy, patient education

## Abstract

Inhaled medications are critical in the pharmaceutical management of respiratory conditions, however, the majority of patients demonstrate at least one critical error when using an inhaler. Since community pharmacists can be instrumental in addressing this care gap, we aimed to determine the rate and type of critical inhaler errors in community pharmacy settings, elucidate the factors contributing to inhaler technique errors, and identify instances when community pharmacists check proper inhaler use. Fourth year pharmacy students on community practice placement (n = 53) identified 200 patients where at least one error was observed in 78% of participants when demonstrating inhaler technique. Prevalent errors of the users were associated with metered dose inhaler (MDI) (55.6%), Ellipta^®^ (88.3%), and Discus^®^ (86.7%) devices. Overall, the mean number of errors was 1.09. Possession of more than one inhaler, use of rescue inhaler, and poor control of asthma were found to be significant predictors of having at least one critical error. In all participating pharmacies, inhaler technique is mainly checked on patient request (93.0%) and for all new inhalers (79.0%).

## 1. Introduction

Inhaled medications are critical in the pharmaceutical management of respiratory conditions such as asthma and chronic obstructive pulmonary disease (COPD). Unfortunately, up to 92% of patients demonstrate at least one critical error when using an inhaler, which can lead to decreased effectiveness of the inhaled drug [1]. Alarmingly, inhaler technique has not improved in the past 40 years [2]. This lack of progress is likely compounded by recent advances in inhaler technologies, which has resulted in a plethora of marketed inhaler devices [3]. Each device requires a different inhalation technique to enable optimal drug delivery to the lungs. The multiple steps required to operate inhalers leaves opportunities for user error and thus, sub-optimal medication efficacy. Indeed, patients who make inhaler technique errors exhibit decreased serum drug levels [4]. Furthermore, inhaler technique errors have been associated with uncontrolled asthma as well as increased rates of severe COPD exacerbations [5,6,7]. Ultimately, poor inhalation technique leads to increased healthcare utilization and costs [8,9].

Numerous studies have attempted to elucidate the factors associated with inhaler technique errors. Potential determinants of correct inhaler use include a recent appointment with a respiratory specialist, using multiple inhalers, novelty of the inhaler, the type of inhaler device, and the age of the patient. Several research groups found that patients who use multiple inhalers commit more errors than those who just use one inhaler [10,11,12]. Unfortunately, the research offers conflicting results. For example, one study found experienced inhaler users committed the most inhaler errors [13], while another study reported that new users had a worse inhaler technique [14]. The relationship between the type of inhaler device and number of inhaler errors is also unclear. Multiple studies report that more patients make errors with dry powder inhalers [10,15], while others suggest that patients make more errors with metered dose inhalers [2,13,16]. The impact of age on proper inhaler technique is another point of contention, however, a recent systematic review favors a negative correlation between older age and proper inhaler technique [5,17,18,19]. 

The discordant results between studies may be due to the extensive differences in the checklists used to score inhaler technique. Evidently, 24 different checklists have been used in various studies to evaluate errors with the Turbuhaler^®^ device, while 16 distinct checklists have been used to assess technique with the Diskus^®^ device [20]. These checklists differed in the number of steps assessed as well as which subset of steps were deemed to be ‘critical’ [20]. An additional complication factor is a vast divergence of how inhaler technique errors, and especially, ‘critical’ errors are defined [9]. Usmani et al. proposed defining a ‘critical’ inhaler error as “an action or inaction that in itself would have a definite detrimental impact on the delivery of the drug to the lung” [9,21]. This is in contrast to a ‘non-critical’ error, which is “an action or inaction that in combination with other factors may, or may not, contribute to ineffective delivery of the drug to the lung” [9]. Many researchers around the world have studied inhaler technique errors and tried to elucidate why mistakes are made. However, research on inhaler technique in Canada is scarce and currently, the rate of patient critical inhaler errors in Alberta is unknown.

Health care practitioners are also contributing to inhaler technique errors and may be inadvertently providing patients with poor technique and instructions [22]. One study found that one in four patients had never received verbal instructions on appropriate inhaler technique [23]. In Canada, many patients do not receive regular counseling on proper inhaler technique [13]. Health care practitioners are failing their patients who suffer from pulmonary conditions. Conversely, studies have also shown that health care practitioners can improve the inhaler technique of their patients and reduce errors [9,18,24,25]. Community pharmacists are particularly situated to regularly check inhaler technique when patients drop off new inhaler prescriptions or pick up refills. Pharmacist interventions can help to improve the inhaler technique of asthma and COPD patients [14,26,27]. Furthermore, patients trust and attempt to follow the pharmacist’s inhaler technique instructions [28]. The patient’s inhaler technique declines over time and thus, regular checks and counseling is required to ensure therapeutic stability [27]. The Global Initiative for Chronic Obstructive Lung Disease—(GOLD) and Global Initiative for Asthma (GINA) guidelines stipulate that inhaler technique needs to be assessed regularly, but do not offer a suggested time frame for re-evaluation [29,30]. One study suggested that pharmacists should conduct inhaler checks every three months to verify proper inhaler technique [27]. However, it is unknown how often pharmacists check inhaler technique in real-life community pharmacy practice.

As a component of a pharmacy student practice based research project, we aimed to:

1. Determine the rate and type of critical inhaler errors in community pharmacy settings in Alberta, Canada.
2. Elucidate the factors contributing to inhaler technique errors.
3. Identify instances when community practice pharmacists check proper inhaler use.

## 2. Materials and Methods

### 2.1. Study Design

This cross-sectional, observational study was conducted between September 2016 and April 2017 in community pharmacies (n = 97) across Alberta, Canada. Fourth year pharmacy students (n = 122) collected data during their eight-week community pharmacy experiential placement. Prior to their placement, students were invited to participate in the study and provided with the necessary resources and training. These final year professional pharmacy students had successfully completed their coursework including therapeutics, pharmacology, and practical casework in the skills lab, which had prepared them to interact with patients with respiratory diseases. In addition, they could rely on the expertise, support, and supervision of their pharmacy preceptors and the pharmacy program study researchers while on their placement.

### 2.2. Pharmacy Students’ Training and Engagement 

Prior to their community placement, students attended an information session, where the study researchers provided details about the study and its rationale, objectives, process of obtaining verbal consent, data collection and reporting, and benefits of their voluntary participation (i.e., research experience and patients’ experience). One week prior to their placement, students were sent a reminder letter about the study and where all necessary resources could be found (i.e., on their online class profile and through their preceptor). The resources included inhaler information, so the students could be reminded of their proper use. Preceptors were informed of the study online and by a letter, which was mailed to the stores together with all necessary forms for the students. 

### 2.3. Inhalation Technique Assessment 

A checklist of critical errors (Table 1) was used to assess inhalation technique and was developed in a two-step process. First, a literature search was performed to identify prior studies aimed at identifying inhaler errors. We identified a cross-sectional observational study by Melani et al. [31] where device errors were identified and classified as either critical errors or non-critical errors. In addition to the observational study, a recent review examining inhaler technique over time was used to create a list of possible inhaler errors for each of the devices [32]. The list of errors was then reviewed and modified with the help of a group of seven experienced Certified Respiratory Educators, each with a minimum of five years’ experience as an Asthma/COPD educator with the Calgary COPD and Asthma program. Appendix A (Table A1) contains a list of devices, producers, and locations. 

### 2.4. Data Collection

To facilitate systematic recording of information, participating students recorded patient information on a structured field notes guide, which was adapted from prior research on the students’ data collection [33]. The following patient variables were collected: patient respiratory diagnosis (asthma, COPD, or other), and whether spirometry was used to diagnose asthma or COPD; for asthma: possession of written asthma action plan (AAP), whether the patient received verbal instructions on how to adjust inhalers when asthma is out of control, asthma control test score, inhaler(s) used and observed errors, if any (using the Critical Inhaler Error Checklist), whether one or more inhalers was new in the last six months, whether the patient had an appointment with a respiratory specialist (physician, nurse, respiratory therapist), and patient’s age. Typical pharmacist practices were also identified. 

The participating pharmacy students were asked to approach up to 15 patients who were 18 years of age or older who were picking up one or more inhaled mediations. Student pharmacists provided a written information letter and verbally explained the study. After verbal informed consent was obtained, patients were asked to demonstrate their inhalation technique with their own device(s) through physical or simulated demonstration and to answer questions about their respiratory condition and care characteristics. The technique assessment results, patient-reported data, and student-reported data were immediately entered in the paper copy or online via the experiential learning management platform. 

### 2.5. Statistical Analysis

Descriptive statistics were used to characterize the number of critical inhaler errors and patient factors (i.e., disease, duration of use, number of devices, and appointments with specialists). For patients with asthma, we were also able to characterize asthma control and the presence of a written or verbal AAP. The relationship between the number of critical errors and patient factors was examined using the appropriate statistical test (e.g., *t*-test, chi-squared test, analysis of variance) with a priori significance level of *p* < 0.05. Statistical software SAS, version 9.3 (Cary, NC, USA) was used to conduct analyses. This study was reviewed and approved by the University of Alberta Health Ethics Research Board. 

## 3. Results

Participating students (n = 53, 43%) recruited a total of 201 patients with data available for 200 patients. Participants were primarily in the age group of 31–50 years (30.5%) and 51–70 years (34%). In terms of diagnosis, the following distribution was noted: asthma n = 126 (63.0%), COPD n = 52 (26.0%), other n = 19 (9.5%), no answer n = 3 (1.5%). The most common device was the metered dose inhaler (MDI)/spacer (55.6%), followed by Turbuhaler^®^ (17%) and Discus^®^ (9.3%), with 45% of participants filling a prescription for two inhalers. Around one-third of participants with asthma (31.7%) reported their condition being controlled (Table 2). 

Almost half of the participants (46.5%) used one device, with a further 45.0% using two devices, and 8.5% using three inhaler devices. Over half of participants (n = 111, 55.5 %) had spirometry, while 38 (19.0%) did not, and 43 (21.5%) were not aware (responses from eight (4%) participants were not applicable). For over a quarter of patients (n = 52, 26.0%), their inhaler(s) were new in the last six months. The majority of participants (n = 127, 63.5%) did not have an appointment with a respiratory specialist in the last six months (Table 3). 

The majority (n = 156, 78%) of the participants had at least one critical error identified when demonstrating inhaler technique, where the mean number of errors was 1.09 (SD = 0.89). Besides the prevalent error of not using a spacer for MDI (74.4%), other common errors were associated with Ellipta^®^, Discus^®^, and Turbuhaler^®^ with a proportion of patients with at least one error at 75%, 66.7%, and 49.1%, respectively. Prevalent errors were similar for all three devices: not checking to see whether the device was empty (Ellipta^®^ = 47.1%, Discus^®^ = 23.3%, Turbuhaler^®^ = 21.8%), not breathing out away from the device (Ellipta^®^ = 35.3%, Discus^®^ = 46.7%, Turbuhaler^®^ = 25.5%). Discus^®^ user errors included not taking a forceful, deep breath (16.7%); Handihaler^®^ users touched the capsule with their fingers when removing it from the device (25%) and did not make the pill rattle when inspiring (20%); Respimat^®^ users did not push the cartridge completely into the inhaler (14.3%) and did not turn the base of the device fully (14.3%) (Table 4). 

The mean number of errors and the proportion of patients with at least one critical error were similar for patients of different age groups, although the oldest group (over 70 years) had the lowest proportion of patients with at least one error (54.2%). The highest proportion was observed in the group of patients aged 31–50 (83.6%) (Table 5). A similar rate and mean of errors were found among patients with asthma and COPD (80.2% and 73.1% with at least one error). The proportion of patients with at least one error was different between participants whose inhaler was new in the last six months vs. not (69.2% vs. 80.0%). Those with more than one inhaler were more likely to demonstrate an error (84.1% vs. 70.9%, *p* < 0.05). Another significant difference in the mean number of errors was observed between rescue and controller inhalers (75.7% vs. 54.6%, *p* < 0.01). There was no observed difference in errors between patients who had a spirometry or who were seen by a respiratory specialist within the last six months compared to those who did not or were not sure. 

For the asthma subgroup, those whose asthma was reported to be controlled had a significantly lower proportion of patients with at least one error compared to the uncontrolled patients (65% vs. 87.1%, *p* < 0.01). There was no significant difference observed for those who had an asthma action plan. 

The students reported that pharmacists in participating pharmacies checked inhaler technique on patient request (93.02%) for all new inhalers (79.07%), while providing care plans or medication management (60.47%); less commonly, the technique was checked for the first refill (27.91%) or for most refills (11.63%). In over one-third of the pharmacies (37.21%), an inhaler technique check was not routine. On average, it took 7.7 minutes to assess inhalation technique. 

## 4. Discussion

Successful mastery of the inhaler technique is a crucial element of asthma and COPD disease management. To support patient inhaled therapy, it is important to identify how often errors occur, who demonstrates errors, and how to intervene. This study aimed to examine the first two aspects of critical inhaler errors in community pharmacy settings in order to identify possible care gaps, and areas for improvement. In line with previous studies [1,14,34], the majority of participants had at least one critical inhaler error and only 10.5% to 31% of patients demonstrated no critical errors. One large-scale study in Italy [31] found that 12% to 43.5% of participants, depending on the device, had at least one critical error. Our finding of prevalent errors were similar to Melani’s study, in other words, not breathing out away from the device (Discus^®^: 43% vs. 46.7%; Turbuhaler^®^: 25% vs. 25.4%) and not taking a forceful, deep breath (Discus^®^: 28% vs. 16.7%; Handihaler^®^ 26% vs. 20%). Some critical errors were not addressed in previous studies such as not using a spacer with a MDI, not checking to see whether the device was empty, touching the capsule with fingers when removing it from the device (specific to Handihaler^®^), or errors associated with devices that were not included in those studies (e.g., Ellipta^®^, Respimat^®^). Studies that did not provide data on specific errors for the studied inhalers [14,34] reported that MDIs, Turbuhaler^®^, and Discus^®^ had the most prevalent critical errors, which is consistent with our findings. 

Among the factors found to be most highly associated with inhaler errors were the number of devices used, controller vs. rescue inhaler, and age. On the other hand, disease, duration of use, spirometry, recent appointment with the specialist, and having a written or verbal AAP were not associated with a decreased likelihood of critical errors. Although having more devices was found to negatively affect the mastery of inhaler use, these findings contribute to the disagreement in the literature [11,31]. When using a rescue inhaler, more patients demonstrated critical errors compared to the controller inhalers, which might be explained by the patients’ low motivation to use a spacer.

In terms of the effect of prior education, we were not able to observe a significant trend in those who did not have a verbal AAP or a written one, which is inconsistent with observations from the study by Melani et al. [31]. In addition, our study found that the age group over 70 years old had the lowest level of critical errors. 

Similar to Melani and colleagues (2011), we found no significant difference in errors between asthmatics and patients with COPD. We also did not observe a significant difference in errors for those whose inhaler was new or not, which is different from Ruud’s study, although both demonstrated frequent errors [14]. A recent appointment with a specialist and knowing whether spirometry was done did not differentiate patients in terms of their rate of critical errors. 

One of the solutions proposed in addressing the high prevalence of critical inhaler errors among patients with chronic respiratory conditions is the active engagement of community pharmacists. They are the most accessible healthcare providers who can effectively identify inhaler technique errors and use it as a source of targeted education [14,34]. Our study confirmed that both new and continuous users of inhalers require attention. In addition, one study suggests that it takes at least three educational sessions by a trained pharmacist to correct inhalation errors [35]. A systematic review of the effectiveness of inhaler technique interventions concluded that the effect decreases over time, which signals the need for periodic reinforcement strategies [36]. 

The present study was a first step in understanding the current state of critical inhaler errors in community pharmacy settings as well as identifying the current practice of community pharmacists in terms of checking inhaler technique. Our results are consistent with those of Braido et al., who found that the act of checking inhaler technique was both selective and rare [37]. Still, research in Alberta suggests that most patients with asthma were asked if they had any questions or concerns about a new prescription in 74% of pharmacies, or concerns about a refill in 40% of pharmacies [38]. Conversations such as these provide opportunities for pharmacists in Alberta and around the world to check on inhaler technique. In our study, pharmacists primarily checked the inhaler technique for new inhalers and when requested by patients. However, only 11–28% of pharmacists checked the inhaler technique for patients with refills. We also aimed to shed more light on the factors associated with poor inhaler technique, as this is still not conclusive. The following future directions and needs were identified: (1) All patients, regardless of the length of time using inhalers, should be checked for inhaler technique; (2) Continuous and regular check-ins are needed for those who demonstrate errors; (3) Barriers to the recommendation for and use of spacers is needed to address this gap, and (4) Checking inhaler technique must be a required step in reimbursed clinical services such as care plans and medication reviews as it takes on average less than eight minutes. 

### Limitations

As previously noted, our checklist did not include all possible inhaler errors; thus, some errors may not have been captured. In addition, the percentage of patients using some devices was quite small, limiting the strength of observed data regarding errors and its association with other factors. Inhaler technique was assessed by the fourth year students who were not otherwise trained to identify errors except in their required respiratory module and corresponding skills lab, which could have introduced interrater variability based on the individual student’s knowledge and experiences.

## 5. Conclusions

In this study, we found that the majority of patients showed at least one critical error when demonstrating inhaler technique. Not using a spacer for MDI, not checking whether the inhaler was empty, and not breathing out away from the device were found to be the most common errors. We also found that the instances with a higher likelihood of inhaler errors (i.e., using a rescue vs. a controller inhaler) and having uncontrolled asthma were situations where negative patient outcomes are more common. Most community pharmacies check inhaler technique upon patient request and for new inhalers, but less commonly when providing care plans or refills. Community pharmacists should be more proactive in monitoring ongoing inhaler use including the use of spacers among patients with respiratory conditions as many of them demonstrate critical errors.

## Figures and Tables

**Table 1:** Types of critical inhaler errors.

Inhaler Device	Critical Error	Error Number
1. Handihaler	Patient swallows the capsule	1
	Did not load the capsule into the device	2
	Did not puncture the capsule	3
	Did not release the puncture needle prior to inspiring	4
	Did not make the pill rattle when inspiring (low inspiratory effort/ability)	5
	Touched the capsule with fingers when removing it from the device	6
2. Breezhaler	Patient swallows the capsule	7
	Did not load the capsule into the device	8
	Did not puncture the capsule	9
	Did not release the puncture needle prior to inspiring	10
	Did not make the pill rattle when inspiring (low inspiratory effort/ability)	11
	Touched the capsule with fingers when removing it from the device	12
3. Turbuhaler	Did not check if the device was empty	13
	Did not take the cap off	14
	Did not twist the colored grip to load the device	15
	Did not breath out away from the device	16
	Did not take a forceful, deep breathe	17
	Tipped device over before inhaling thereby emptying out some of the medication	18
4. Diskus	Did not check if the device was empty	19
	Did not open the device	20
	Did not load the device	21
	Did not breath out away from the device	22
	Did not take a forceful, deep breathe	23
	Tipped device over before inhaling thereby emptying out some of the medication	24
5. Genuair	Did not check if the device was empty	25
	Did not take the cap off	26
	Did not push and release the colored button	27
	Patient inhaled when the colored control window was red NOT green	28
	Can’t actuate the device	29
	Did not know the device was empty	30
6. Ellipta	Did not check to see if the device was empty	31
	Did not open the device completely	32
	Tipped the device horizontally so that medication is lost prior to inhalation	33
	Did not breath out away from the device	34
	Did not close the lid of the device until its clicks closed at the end of use	35
7. Respimat	Did not insert the cartridge into the inhaler	36
	Did not push the cartridge completely into the inhaler	37
	Did not turn the base of the device fully (until click heard) (T= turn)	38
	Did not remove the cap (O = open)	39
	Did not press the button to release medication (P = press)	40
8. MDI + spacer	Did not use the spacer	41
	Did not take off cap	42
	Teeth or lips block the spacer mouthpiece	43
	Use of incorrect mask size (for CPAS)	44
9. Any device	Used expired medication	45

A critical error represents an error that causes the patient to receive zero/reduced drug from the 4device. This list represents some, but not all of the critical inhaler errors. REMINDER: A patient may still have INADEQUATE technique without identifying a CRITICAL error.

**Table 2:** Participants’ characteristics (n = 200 participants).

Characteristics	n (%)
Age (years)	
18–30	45 (22.5)
31–50	61 (30.5)
51–70	68 (34.0)
>70	24 (12.0)
No answer	2 (1.0)
Diagnosis	
Asthma	126 (63.0)
COPD	52 (26.0)
Other	19 (9.5)
No answer	3 (1.5)
Number of inhaler devices	
1	93 (46.5)
2	90 (45.0)
3	17 (8.5)
Asthma Control Test Score ^1^ (n = 126 with asthma)	
0	40 (31.7)
1	29 (23.0)
2	22 (17.5)
3	14 (11.1)
4	15 (11.9)
5	5 (4.0)
Missing Data	1 (0.8)

^1^ A score of one or higher indicates that asthma may not be controlled.

**Table 3:** Respiratory management (n = 200).

Respiratory Management Characteristic	Yes	No	Don’t Know	No Answer
	n (%)			
All Participants				
Spirometry testing	111 (55.5)	38 (19.0)	43 (21.5)	8 (4.0)
New inhaler in the last 6 months	52 (26.0)	140 (70.0)	4 (2.0)	4 (2.0)
Appointment with the specialist in the last 6 months	59 (29.5)	127 (63.5)	13 (6.5)	1 (0.5)
Participants with Asthma (n = 126)				
Written Asthma Action Plan	17 (13.5)	99 (78.6)	10 (7.9)	-
Verbal Asthma Action Plan	77 (61.1)	41 (32.5)	7 (5.6)	1 (0.8)

**Table 4:** Critical errors by inhaler type (n = 324 inhalers reported).

Inhaler	Users, n	Total Critical Errors, n (%)	Error Type, n (%)	Users with at Least One Error, n (%)
Handihaler^®^	19	13 (65)	4-Did not puncture: 2 (10)	8 (42.1)
			5-No rattle: 4 (20)	
			6-Touched capsule: 5 (25)	
			41-No spacer: 2 (10)	
Breezhaler^®^	7	2 (28.6)	11-No rattle: 1 (14.3)	1 (14.3)
			12-Touched capsule: 1 (14.3)	
Turbuhaler^®^	55	35 (63.6)	13-No check if empty: 12 (21.8)	27 (49.1)
			15-No twist: 2 (3.6)	
			16-No out breath: 14 (25.5)	
			17-No deep breath: 6 (10.9)	
			18-Tipped device: 1 (1.8)	
Discus^®^	30	26 (86.7)	19-No check if empty: 7 (23.3)	20 (66.7)
			22-No out breath: 14 (46.7)	
			23-No deep breath: 5 (16.7)	
Genuair^®^	1	2 (100)	25-No check if empty: 1 (50)	1/1 (100)
			29-No actuation: 1 (50)	
Ellipta^®^	16	15 (88.3)	31- No check if empty: 8 (47.1)	12 (75)
			33-Tipped device: 1 (5.9)	
			34-No out breath: 6 (35.3)	
Respimat^®^	12	4 (28.6)	37-Did not push the cartridge: 2 (14.3)	4 (33.3)
			38-Did not fully turn base: 2 (14.3)	
MDI + Spacer	151	140 (77.8)	41-No spacer: 134 (74.4)	119 (78.8)
			42-Did not remove cap: 1 (0.6)	
			43-Blocked mouthpiece: 5 (2.8)	

**Table 5:** Critical inhaler errors and associated characteristics.

Characteristic	At Least One Critical Error, n (%)	*p*-Value
**Disease (n = 197)**		
asthma	101/126 = 80.2	0.53
COPD	38/52 = 73.1	
other	14/19 = 73.7	
**Duration of use**		
new	36/52 = 69.23	0.11
old	112/140 = 80	
**Number of devices**		
one	66/93 = 70.97	0.025
two–three	90/107 = 84.11	
**Controller (yes no)^1^**		
controller	83/152 = 54.6	0.0002
rescue	109/144 = 75.7	
**Spirometry**		
yes	86/111 = 77.5	0.499
no/don’t know	66/81 = 81.5	
**Appointment with specialist**		
yes	46/59 = 77.97	0.99
no	99/127 = 77.9	
**Asthma control:**		
controlled (0)	26/40 = 65	0.004
uncontrolled (1–5)	74/85 = 87.1	
**Written AAP**		
yes	14/17 = 82.3	0.81
no/don’t know	87/109 = 79.8	
**Verbal AAP**		
yes	60/77 = 77.9	0.30
no/don’t know	41/48 = 85.4	
**Age**		
1 = 18–30	35/45 = 77.8	0.019 ^2^
2 = 31–50	51/61 = 83.6	
3 = 51–70	56/68 = 82.3	
4 ≥ 70	13/24 = 54.2	

^1^ n = 295 (n = 152 controller, and n = 144 rescue); ^2^ Tukey’s post-hoc analyses of mean number of errors revealed significant difference between groups 3 and 4.

## Appendix A

**Table A1:** Manufacturer information for products used in the study.

Inhaler Device	Manufacturer
1. Handihaler	Boehringer Ingelheim (Canada) Ltd./Ltee.
	Burlington, Ontario
	Canada
2. Breezhaler	Novartis Pharmaceuticals Canada Inc.
	Dorval, Quebec
	Canada
3. Turbuhaler	Astrazeneca Canada Inc.
	Mississauga, Ontario
	Canada
4. Diskus	GlaxoSmithKline Inc.
	Mississauga, Ontario
	Canada
5. Genuair	Astrazeneca Canada Inc.
	Mississauga, Ontario
	Canada
6. Ellipta	GlaxoSmithKline Inc.
	Mississauga, Ontario
	Canada
7. Respimat	Boehringer Ingelheim (Canada) Ltd./Ltee.
	Burlington, Ontario
	Canada
8. MDI + spacer	Valeant Canada Lp/Valeant Canada S.E.C.
	Laval, Quebec
	Canada
	+
	Trudell Medical International
	London, Ontario
	Canada

Information available from Health Canada Drug Project Database [39].
